# Associations between perinatal HIV-related risk factors and select serum PUFA levels among Ugandan children and adolescents

**DOI:** 10.1017/S1368980025100451

**Published:** 2025-07-01

**Authors:** Vanessa N. Cardino, Selin Sergin, Sarah K. Zalwango, Jenifer I. Fenton, Amara E. Ezeamama

**Affiliations:** 1 Department of Food Science and Human Nutrition, Michigan State University, 469 Wilson Road, East Lansing, MI 48824, USA; 2 Kampala Capital City Authority, City Hall, Plot 1-3, Apollo Kaggwa Road PO BOX 7010 Kampala, Uganda; 3 Department of Psychiatry, Michigan State University, 909 Wilson Road, East Lansing, MI 48824, USA

**Keywords:** Uganda, PUFA, Antiretroviral therapy, Children, HIV

## Abstract

**Objective:**

To (1) determine how serum fatty acid (FA) levels differ by developmental stage, (2) quantify associations between perinatal HIV-related factors and PUFA levels and (3) examine the heterogeneity of these associations by developmental stage.

**Design:**

Cross-sectional secondary analysis of baseline data from two prospective cohorts.

**Setting:**

Kampala, Uganda.

**Participants:**

243 children (6–10 years old) and 383 adolescents (11–18 years old) were recruited at Kawaala Health Center based on perinatal HIV status. Youth (children and adolescents) were classified as: those with perinatal HIV infection (PHIV: *n* 212), those perinatally HIV exposed but remained uninfected (HEU: *n* 211) and those perinatally HIV unexposed and uninfected (HUU: *n* 203).

**Results:**

Adolescents had lower *n*-6 and *n*-3 PUFA levels than children, and among adolescents, these levels increased with age. Relative to children HUU, children PHIV had a higher triene:tetraene ratio and 20:3*n*-9 (indicators of essential fatty acid deficiency (EFAD)). Adolescents PHIV *v*. HUU had lower 20:5*n*-3 levels. When considering *in utero*/peripartum antiretroviral therapy (IPA) exposure, the FA profile was indicative of EFAD for youth PHIV with (a) no IPA exposure and (b) combination IPA exposure, whereas non-nucleoside RT inhibitor+nucleoside RT inhibitor exposure was associated with a favourable FA profile among youth PHIV and HEU (all *P* < 0·05).

**Conclusion:**

In this sample, perinatal HIV status was associated with low PUFA levels, and these associations varied by developmental stage and IPA exposure type. Future research should elucidate the contribution of IPA exposure type to EFAD and the implications of these differences on growth and cognitive development.

Essential fatty acids (EFA) are fatty acids (FA) that cannot be synthesised by the human body and therefore must be consumed from the diet^(1)^. The two EFA are the omega-6 (*n*-6) linoleic acid (18:2*n*-6) and the omega-3 (*n*-3) *α*-linolenic acid (18:3*n*-3). 18:2*n*-6 is commonly found in vegetable and safflower oils, and 18:3*n*-3 is abundant in walnuts, flaxseeds, chia seeds and canola and soybean oils^(2)^. The human body can metabolise 18:2*n*-6 and 18:3*n*-3 once ingested into downstream *n*-6 and *n*-3 metabolites^(3)^. Notable metabolites include *n*-6 arachidonic acid (20:4*n*-6) and *n*-3 DHA (22:6*n*-3). These PUFA regulate inflammatory response^(4)^, maintain cell membrane integrity and support brain health^(5)^ and growth^(6,7)^. EPA (20:5*n*-3) is another *n*-3 PUFA that plays a significant role in brain health and inflammation regulation^(8)^. These metabolites are considered ‘conditionally essential’ because conversion efficiency from 18:2*n*-6 and 18:3*n*-3 is low^(9)^. 20:4*n*-6 is found in animal products^(10)^, while 20:5*n*-3 and 22:6*n*-3 are present in fatty fish^(11)^. Only 18:2*n*-6 and 18:3*n*-3 have adequate intake levels, which vary from 9–18 g/d and 1·0–1·7 g/d, respectively, and depend on age and sex^(12)^. While dietary sufficiency of *n*-6 and *n*-3 PUFA is important throughout the lifespan, it is especially critical during periods of rapid growth and development, such as childhood and adolescence.

To date, there is little empirical understanding of risk factors associated with PUFA insufficiency. EFA deficiency (EFAD) is characterised metabolically as an increased production of the omega-9 (*n*-9) PUFA mead acid (20:3*n*-9) (triene) and a decreased production of 20:4*n*-6 (tetraene). Hence, the triene:tetraene (T:T) ratio is clinically used as a biomarker for EFAD^(13)^. The cut point for diagnosing EFAD is a T:T ratio greater than 0·02 based on levels seen in individuals with known fat malabsorption^(14)^. Some researchers, however, believe it should be more conservative at greater than 0·2^(15)^ since this cut point is associated with more severe symptoms such as dry skin, hair loss, growth retardation and reduced brain weight^(16)^. Nevertheless, monitoring T:T ratio levels is a helpful strategy to assess insufficiency of EFA intake in human populations vulnerable to low PUFA intake.

Individuals infected with or exposed to HIV may be especially vulnerable to insufficient PUFA levels due to both malnutrition and malabsorption. General malnutrition is prevalent among individuals affected by HIV^(17)^, especially youth (i.e. children and adolescents)^(18)^. Malnourished Ugandan youth infected with HIV have lower SFA, higher MUFA and lower total *n*-6 and *n*-3 PUFA levels^(19)^. Malnutrition and low PUFA levels may partially result from impaired nutrient absorption due to HIV-associated intestinal dysbiosis. Youth with perinatal HIV infection have less faecal beta diversity and relative abundance compared to children who were perinatally exposed to HIV but remained uninfected, but both groups had similar microbiome profiles, gut damage, microbial translocation^(20)^ and inflammation marker levels^(21)^. These observations are partly explained by the fact that HIV infection primarily affects CD4 + T cells in the gut-associated lymphoid tissue that help colonise gut bacteria and likely influences intestinal health^(22)^.

Additionally, youth with perinatal HIV infection or exposure may be more susceptible to low PUFA levels than youth without perinatal HIV infection or exposure due to their exposure to *in utero/*peripartum antiretroviral therapy (IPA). Healthcare providers administer IPA to prevent the vertical transmission of HIV from mother to child. While IPA and current antiretroviral therapy (ART) may reduce dysbiosis by restoring gut-associated lymphoid tissue lymphocytes, studies among adults show this restoration is slow and often does not completely eliminate dysbiosis^(22,23)^. The type of IPA regimen to which individuals are exposed matters, with protease inhibitor- and nucleoside RT inhibitor (NRTI)-based ART associated with more gut dysbiosis, immune activation and inflammation relative to other forms of ART such as non-nucleoside RT inhibitors (NNRTI)^(24,25)^. Hence, perinatal HIV-related factors may result in malnutrition and malabsorption, but their relevance for PUFA levels is largely unknown.

Ugandan youth may be especially vulnerable to low PUFA intake because of their dietary patterns and high incidence of perinatal HIV exposure and/or infection^(26–28)^. The traditional Ugandan diet is high in starchy vegetables and grains and low in oils and animal products, thus low in dietary fat^(29,30)^. Recent westernisation of the Ugandan diet has led to lower intake of PUFA, demonstrated in Ugandan adults^(31)^ and adolescents^(26,27)^ but not children. Additionally, over 150 000 Ugandan youth are living with HIV, and over 1 000 000 Ugandan children were perinatally HIV exposed but remained uninfected^(28)^. Consequently, Ugandan youth, particularly adolescents, may be susceptible to PUFA deficiencies.

Thus, the objectives of this study are to (1) determine how serum FA levels differ by developmental stage, (2) quantify associations between perinatal HIV-related factors and PUFA levels and (3) examine the heterogeneity of these associations by developmental stage. It was expected that FA levels would differ between children and adolescents, with adolescents having higher SFA levels and lower PUFA levels. Additionally, HIV severity and lack of IPA exposure were expected to be negatively associated with PUFA levels, with these associations varying by developmental stage due to differences in temporal proximity to HIV exposure and/or infection.

## Methods

### Study design and participants

The current study is a cross-sectional secondary analysis of baseline youth FA, demographic and clinical data from two prospective cohort studies. Youth (i.e. those 6–18 years old; *n* 772) from Kampala, Uganda were enrolled for two prospective cohort studies of differences in functional survival and developmental trajectory by perinatal HIV status. The first study recruited children (i.e. those 6–10 years old; *n* 305) who were enrolled from March 2017 to September 2018^(32)^. The second study recruited adolescents (i.e. those 11–18 years old; *n* 467) who were enrolled between October 2018 and December 2019^(33)^. The total sample included those with perinatal HIV infection (PHIV: *n* 259), those perinatally HIV exposed but remained uninfected (HEU: *n* 257) and control participants perinatally HIV unexposed and uninfected (HUU: *n* 256). At enrollment, study personnel collected socio-demographic information and blood samples from, and performed health assessments on, both participants and their caregivers.

### Recruitment

All study participants were recruited from the Kawaala Health Center (KHC), and the recruitment procedure varied according to perinatal HIV status. As previously described^(7)^, youth PHIV were recruited from current patients receiving HIV treatment at KHC. Youth HEU were recruited through the medical records of adult women infected with HIV cared for at KHC and the Early Infant Diagnosis Registry. Youth HUU were recruited from KHC’s outpatient department or referral from already-enrolled subjects.

### Eligibility criteria

Participants were included in the cohort studies if they were 6–18 years old at the time of enrollment, had available health records with objective data on their birth, including HIV status of their biological mother and IPA exposure and provided a viable serum sample (i.e. at least 200 uL without hemolysis). Participants were excluded from the study if they were born in a non-clinic setting and had no antenatal register or delivery medical records.

Caregivers could participate in the cohort studies if they were at least 18 years old at the time of enrollment and provided an affirmative response confirming that they were the primary caregiver for a study-eligible child for at least 6 months prior to enrollment.

### Primary potential risk factors: HIV-related factors

Perinatal HIV status: Perinatal HIV status for youth PHIV and HEU was determined by a positive or negative DNA PCR test, respectively, before 18 months of age. At enrollment, the HIV status of youth HEU and HUU was confirmed by a negative HIV rapid diagnostic test.

IPA exposure status: IPA status was identified through medical records and categorised separately for youth PHIV and HEU. The study base includes youth born to women living with HIV in the pre-option B+ era when combination ART (cART) in pregnancy was only given to very sick mothers, and IPA type varied per prevailing standard of care^(34–36)^. IPA exposure types include: (1) no exposure, (2) single-dose nevirapine (a non-nucleoside RT inhibitor (NNRTI)) with or without zidovudine (a nucleoside RT inhibitor (NRTI)) (sdNVP ± ZDV), (3) single-dose nevirapine with zidovudine and lamivudine (a NRTI) (sdNVP + ZDV + 3TC) and (4) cART, which is a combination of more than two ART drug classes that were given to mothers who had low enough CD4 + T cell counts for access. All HUU had no IPA exposure, so there are nine categories in total (Table 1).

**Table 1.** List of HIV-related factor categories with abbreviations and explanations

Perinatal HIV categories	Abbreviation	IPA exposure	Drug class	Abbreviation	IPA exposure categories
Perinatally HIV unexposed and uninfected^*^	HUU	No *in utero*/peripartum antiretroviral therapy exposure	N/A	No IPA	HUU^*^
Perinatally HIV exposed but remained uninfected	HEU	Single-dose nevirapine	Non-nucleoside RT inhibitor	sdNVP	HEU w/ no IPA
With perinatal HIV infection	PHIV	Zidovudine	Nucleoside RT inhibitor	ZDV	HEU w/ sdNVP ± ZDV
		Lamivudine	Nucleoside RT inhibitor	3TC	HEU w/ sdNVP + ZDV + 3TC
		Combination ART	Two or more drug classes	cART	HEU w/ cART
					PHIV w/ no IPA
					PHIV w/ sdNVP ± ZDV
					PHIV w/ sdNVP + ZDV + 3TC
					PHIV w/ cART

IPA, *in utero/*peripartum antiretroviral therapy; HUU, perinatally HIV unexposed and uninfected; HEU, perinatally HIV exposed but remained uninfected; PHIV, with perinatal HIV infection.

* Reference group.

Youth PHIV risk factors: CD4 nadir, which is the lowest CD4 + T cell count (measured as cells/ml) recorded since initiation of treatment at KHC, was determined from medical records and classified into three groups: (1) < 250 cells/ml (2) 250–500 cells/ml (3) > 500 cells/ml. Current ART was identified during the baseline health assessment and classified using the following categories: (1) cART naïve or unspecified, (2) cART containing nevirapine (NVP), (3) cART containing NVP and a NRTI (either abacavir or tenofovir), (4) cART containing Kaletra (a protease inhibitor- adolescents only) and (5) cART containing dolutegravir (DTG), an integrase inhibitor.

### Secondary potential risk factors

Age (in years), biological sex (male *v*. female) and developmental stage (child *v*. adolescent) were determined through the baseline interview. Caregiver age, sex and years of education were provided by caregivers to study personnel upon enrollment. Caregiver HIV status was extracted from caregiver medical records. All these variables besides caregiver HIV status were considered potential confounders.

### Other characteristics

Height-for-age z-score was calculated from the youth’s height at baseline (measured in inches with a wall ruler) using WHO AnthroPlus. BMI-for-age z-score was determined from the youth’s height and weight (measured in kilograms on a calibrated beam balance scale (Seca Classic Beam Scale, Model#700, Seca Inc) using WHO AnthroPlus. Height-for-age z-score categories were created based on stunting risk status, and BMI-for-age z-score categories were created based on deviation from normal weight. Household food spending (i.e. the amount of money spent on food per day in Ugandan shillings) was provided by caregivers upon enrollment.

### Primary outcome: serum fatty acid levels

Study personnel at KHC collected venous blood from fasted participants at the time of enrollment and separated the serum fractions for storage at –80°C. The serum samples were deidentified and shipped to Michigan State University for FA methylation and quantification.

### Serum fatty acid methylation

FA were extracted from serum and methylated for GC/MS analysis based on the methods of Glaser, Demmelmair and Koletzko using HPLC-grade reagents purchased from Sigma-Aldrich unless stated otherwise^(37)^. Briefly, 250 uL of each serum sample and 5 ug of internal standard stearic acid-d35 (Cayman Chemical) dissolved in 50 uL isooctane were vortexed with 1·5 ml of 1:4 (v/v) methanol:acetyl chloride (0·1 % w/v butylated-hydroxy-toluene). All samples were then heated to 100℃ for 1 h and 15 min to promote methylation of FA. After the tubes cooled to room temperature, 2 ml of 5 % (w/v) sodium bicarbonate was added to each tube to neutralise methylation. The tubes received two cycles of 2 ml of hexane, a 30-sec shaking and centrifugation at 2500 RPM for 5 min at room temperature to dissolve the non-polar FA methyl esters (FAME). The hexane layers containing FAME were pooled and dried under nitrogen. The FAME were dissolved in 250 ul of isooctane. 200 ul were aliquoted into GC vials for GC/MS analysis, and the vials and remaining solutions were stored at –20℃ until GC/MS analysis.

### Serum fatty acid quantification

A Clarus 600/680 gas chromatograph/mass spectrometer (Perkin-Elmer) with an HP-88 column (100 m length, 0·25 mm internal diameter, 0·2 µm film thickness) (Agilent) and helium carrier gas was used to quantify the FA within each sample. The GC method was adapted from Criado-Navarro et al.^(38)^ Specifically, the GC was held at 80℃ for 5 min, ramped to 240℃ at a rate of 4℃/min and held for 11 min. MS data were generated using full scan mode with a mass/charge ratio range of 70–400. FA were quantified using an external standard curve made from Supelco 37-component FAME mix and individual FAME standards for 16:1t, 20:3*n*-9, 22:4*n*-6, 22:5*n*-3 and 22:5*n*-6. (Cayman Chemical). The resulting chromatograms were uploaded to TargetLynx version 4.0.1 (Waters Corporation) for peak integration, which resulted in concentrations and proportions for each FA measured. Twenty-three FA were quantified for all participants as a percentage of total FA (% total FA). The validity of quantifying serum FA to reflect dietary FA intake is supported by various studies^(6)^.

### Serum fatty acid sum/ratio calculations and key measures

Total SFA level was calculated by adding all SFA measured (14:0, 16:0, 18:0, 20:0, 22:0 and 24:0). Total MUFA level was calculated by summing all measured MUFA (16:1c7 + 16:1c9, 16:1t, 18:1c9 + 18:1c11, 20:1*n*-9 and 24:1*n*-9). Total *n*-6 PUFA level includes all *n*-6 PUFA measured (18:2*n*-6, 18:3*n*-6, 20:2*n*-6, 20:3*n*-6, 20:4*n*-6, 22:4*n*-6 and 22:5*n*-6). Total *n*-3 PUFA level sums all *n*-3 PUFA measured (18:3*n*-3, 20:5*n*-3, 22:5*n*-3, 22:6*n*-3). Omega-3 Index is the sum of red blood cell 20:5*n*-3 and 22:6*n*-3 levels (calculated from serum 20:5*n*-3 and 22:6*n*-3 using the equation: (% serum 20:5*n*-3+% serum 22:6*n*-3 + 0·0141)/0·7799^(39)^. Total PUFA level is the sum of all *n*-3 PUFA, *n*-6 PUFA and 20:3*n*-9. Total highly unsaturated FA (HUFA) level was calculated by summing 20:3*n*-6, 20:4*n*-6, 20:5*n*-3, 22:4*n*-6, 22:6*n*-3, 22:5*n*-3, 22:5*n*-6 and 20:3*n*-9. HUFA ratio is the ratio of all *n*-3 HUFA over total HUFA times 100^(4)^. The T:T ratio is the ratio of triene (20:3*n*-9) to tetraene (20:4*n*-6). Key FA measures included T:T ratio, 20:3*n*-9, 18:2*n*-6, 20:4*n*-6, 18:3*n*-3, 20:5*n*-3 and 22:6*n*-3 because of their implications for EFA sufficiency^(4–9,13)^. Markers of PUFA insufficiency include a higher T:T ratio and 20:3*n*-9 levels and lower 18:2*n*-6, 20:4*n*-6, 18:3*n*-3, 20:5*n*-3 and 22:6*n*-3 levels^(4–9,13)^.

### Statistical analysis

A cross-sectional analysis of baseline data from the two prospective cohort studies was performed, and participants with missing FA data were excluded from the analysis. Specifically, statistical analyses included descriptive and multivariable analyses implemented in R and Stata. Descriptive statistics were computed in the overall sample and according to developmental stage. These statistics included mean differences with corresponding sd for continuous variables calculated via two-tailed Student’s *t* test and differences in proportion with corresponding counts for categorical risk factor variables calculated using Chi-squared or Fisher’s exact tests. Additional descriptive analyses estimated means and sd for all FA levels, sums and ratios overall as well as differences in mean value by developmental stage. Separate Pearson correlations were calculated for children and adolescents to yield correlation matrices to observe correlations between T:T ratio and select PUFA levels. The R package ‘corrplot’ was used to generate the correlation matrices. Statistical significance was determined at *α* = 0·05.

To observe relationships between risk factor variables and select continuous PUFA levels and ratios among all study participants, multivariable linear regression models clustered by household were implemented in Stata to estimate beta (*ß*) values and 95 % CI for children and adolescents separately. Children and adolescents were analysed separately because their overall FA profiles differed significantly (see Table 3), making developmental stage an effect modifier. These models were mutually adjusted for the following confounders: age, sex, perinatal HIV status, caregiver sex and caregiver education years. Similar models were run separately for children and adolescents PHIV to study associations between current HIV treatment factors (CD4 nadir, current ART) and select continuous PUFA levels and ratios. These models were adjusted for the following confounders: age, sex, caregiver sex and caregiver education years. Participants were not included in these models if they had missing data for any confounders.

**Table 2.** Baseline characteristics by developmental stage among 6–18-year-old Ugandan youth

	Participants with missing data	Total (*n* 626)		Children (6–10 years old) (*n* 243)		Adolescents (11–18 years old) (*n* 383)		
Characteristic		Mean	sd	Mean	sd	Mean	sd	*P*-value
Youth characteristic								
Age (years)	1	11·5	3·6	7·7	1·5	13·9	2·3	N/A
		*n*	%	*n*	%	*n*	%	
Sex	0							0·06
Male		299	48	128	53	171	45	
Female		327	52	115	47	212	55	
Caregiver characteristic		Mean	sd	Mean	sd	Mean	sd	
Caregiver age (years)	9	39·1	11·6	34·5	8·6	41·9	12·4	< 0·001***
Caregiver education (years)	55	6·4	3·8	6·7	3·8	6·3	3·8	0·15
Money spent on food per day (Ugandan schillings)	13	11 060	6043	11 000	6336	11 096	5862	0·85
		*n*	%	*n*	%	*n*	%	
Caregiver sex	30							0·07
Male		68	11	19	8	49	13	
Female		528	89	213	92	315	87	
HIV-related factors		*n*	%	*n*	%	*n*	%	
Perinatal HIV status	0							0·44
HUU		203	32	81	33	124	32	
HEU		211	34	78	32	133	35	
PHIV		212	33	84	35	126	33	
IPA exposure	2							0·79
HUU		205	33	81	34	124	32	
HEU								
None		94	15	37	15	57	15	
sdNVP 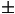 ZDV		45	7	19	8	26	7	
sdNVP + ZDV + 3TC		34	5	8	3	26	7	
cART		37	6	13	5	24	6	
PHIV								
None		110	18	46	19	64	17	
sdNVP 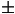 ZDV		21	3	9	4	12	3	
sdNVP + ZDV + 3TC		21	3	8	3	13	3	
cART		57	9	20	8	37	10	
Caregiver HIV status	8							0·69
HIV-		223	36	88	37	135	35	
HIV+		395	64	148	63	247	65	
If PHIV		*n*	%	*n*	%	*n*	%	
CD4 nadir	30							0·02*
< 250 cells/ml		29	16	12	16	17	16	
250–500 cells/ml		55	30	14	19	41	38	
> 500 cells/ml		98	54	48	65	50	46	
Current ART exposure	6							< 0·001^***†^
cART-naïve/unknown		60	29	28	33	32	27	
NVP		52	25	41	49	11	9	
NVP + NRTI		14	8	11	13	6	5	
Kaletra		70	34	0	0	70	57	
DTG		7	3	4	5	3	2	
Malnutrition-related growth outcomes		*n*	%	*n*	%	*n*	%	
BMI-for-age z-score	6							0·24
Underweight (BAZ < –1·5)		109	18	47	20	62	16	
Normal weight (–1·5 ≤ BAZ ≤ 1·5)		497	80	187	79	310	81	
Overweight (BAZ > 1·5)		14	2	3	1	11	3	
Height-for-age z-score	0							< 0·001***
No evidence of stunting (HAZ > –1·5)		440	70	199	82	241	62	
At risk of stunting (–2 ≤ HAZ ≤ –1·5)		93	15	25	10	68	18	
Stunted (HAZ < –2)		93	15	19	8	19	20	

HUU, perinatally HIV unexposed and uninfected; HEU, perinatally HIV exposed but remained uninfected; PHIV, with perinatal HIV infection; IPA, *in utero*/peripartum antiretroviral therapy; sdNVP, single-dose nevirapine; ZDV, zidovudine; 3TC, lamivudine; cART, combination antiretroviral therapy; ART, antiretroviral therapy; NVP, nevirapine; NRTI, nucleoside RT inhibitor; DTG, dolutegravir; BAZ, BMI-for-age z-score; HAZ, height-for-age z-score.

*P < 0·05, ***P < 0·001.

† Calculated using Fisher’s exact test.

## Results

### Baseline characteristics by developmental stage

Of the 772 children and adolescents recruited for the prospective cohort studies, 626 provided viable serum samples and thus were included in this cross-sectional analysis. The total study population of 626 children and adolescents had a mean age of 11·5 (sd: 3·6) years and was 52 % female (Table 2). The mean age of children was 7·7 years (sd: 1·5), and the mean age of adolescents was 13·9 years (sd: 2·3). Caregivers of children were significantly younger than caregivers of adolescents (mean (sd): 34·5 (8·6) years *v.* 41·9 (12·4) years, *P* < 0·001). Among participants PHIV, 16 % had CD4 nadir levels < 250 cell/ml, and this exposure was similar for children and adolescents. However, CD4 nadir levels > 500 cells/ml occurred more frequently in children (65 %) compared with adolescents (46 %) (*P* = 0·02). Most children PHIV (62 %) were on NVP-based cART, and the majority of adolescents (57 %) were on Kaletra-based cART (*P* < 0·001). Stunting or at risk of stunting was more prevalent in adolescents relative to children (38 % *v.* 18 %, *P* < 0·001).

### Fatty acid levels by developmental stage

**Figure 1.**
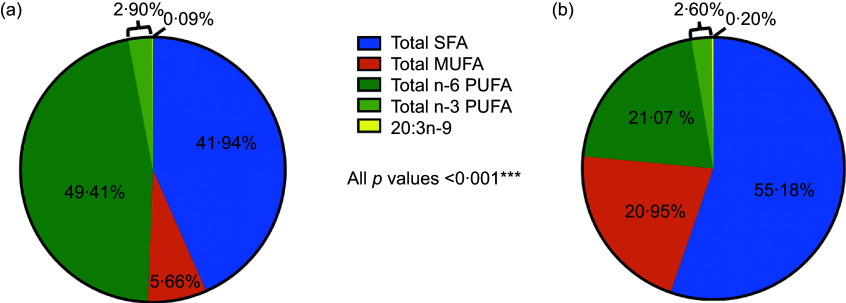
Serum fatty acid levels reported as % total fatty acids.

The average levels of all FA and EFAD prevalence differed significantly by developmental stage (all *P* < 0·001) (Table 3). Overall, children had healthier FA profiles compared with adolescent peers including: lower SFA (41·94 % *v.* 55·18 %), MUFA (5·66 % *v.* 20·95 %) and 20:3*n*-9 levels (0·09 % *v.* 0·20 %) and higher *n*-6 PUFA (49·41 % *v.* 21·07 %) and *n*-3 PUFA (2·90 % *v.* 2·60 %) levels (all *P* < 0·001) (Figure 1).

**Table 3.** Serum fatty acid levels, sums and ratios among 6–18-year-old Ugandan youth by developmental stage

		Total (*n* 626)		Children (*n* 243)		Adolescents (*n* 383)		
FA class	FA formula	Mean %^†^	sd	Mean %	sd	Mean %	sd	*P*-value
SFA	14:0	0·78	0·30	0·67	0·34	0·86	0·26	< 0·001***
	16:0	34·05	3·90	29·19	3·30	37·13	2·78	< 0·001***
	18:0	12·75	2·41	11·00	2·12	13·86	1·86	< 0·001***
	20:0	0·43	0·21	0·20	0·07	0·58	0·12	< 0·001***
	22:0	1·07	0·53	0·58	0·22	1·39	0·42	< 0·001***
	24:0	0·96	0·60	0·32	0·13	1·37	0·39	< 0·001***
MUFA	16:1c7 + 16:1c9	1·52	0·65	1·03	0·50	1·83	0·52	< 0·001***
	16:1t	0·11	0·06	0·07	0·04	0·13	0·06	< 0·001***
	18:1c9 + 18:1c11	11·65	6·85	3·40	0·78	16·89	2·35	< 0·001***
	20:1*n*-9	0·31	0·20	0·36	0·29	0·28	0·09	< 0·001***
	24:1*n*-9	1·42	0·66	0·80	0·25	1·82	0·53	< 0·001***
*n*-6 PUFA	18:2*n*-6	22·49	11·98	36·73	5·37	13·45	2·37	< 0·001***
	18:3*n*-6	0·13	0·07	0·06	0·05	0·17	0·05	< 0·001***
	20:2*n*-6	0·27	0·09	0·20	0·08	0·31	0·07	< 0·001***
	20:3*n*-6	1·29	0·68	0·56	0·26	1·76	0·39	< 0·001***
	20:4*n*-6	7·20	4·02	11·81	2·29	4·28	1·03	< 0·001***
	22:4*n*-6	0·49	0·43	0·02	0·02	0·78	0·26	< 0·001***
	22:5*n*-6	0·21	0·19	0·01	0·01	0·33	0·13	< 0·001***
*n*-3 PUFA	18:3*n*-3	0·29	0·14	0·26	0·16	0·31	0·13	< 0·001***
	20:5*n*-3	0·41	0·23	0·46	0·33	0·38	0·14	< 0·001***
	22:5*n*-3	0·40	0·33	0·07	0·04	0·61	0·25	< 0·001***
	22:6*n*-3	1·61	0·83	2·11	0·90	1·30	0·60	< 0·001***
Sums and ratios	20:5*n*-3 + 22:6*n*-3	2·03	0·98	2·57	1·13	1·68	0·67	< 0·001***
	Omega-3 Index	2·61	1·25	3·31	1·45	2·17	0·86	< 0·001***
	Total PUFA	34·94	14·50	52·39	5·07	23·87	3·33	< 0·001***
	*n*-6:*n*-3 ratio	13·40	9·60	20·57	11·93	8·86	2·69	< 0·001***
	Total HUFA	11·77	3·74	15·12	3·14	9·64	2·22	< 0·001***
	HUFA ratio	20·98	6·08	17·04	5·06	23·48	5·30	< 0·001***
EFAD biomarkers	20:3*n*-9	0·15	0·11	0·09	0·06	0·20	0·12	< 0·001***
	T:T ratio	0·03	0·03	0·01	0·00	0·05	0·03	< 0·001***
		*n*	%	*n*	%	*n*	%	
	EFAD prevalence^‡^	355	57	3	0·01	352	92	< 0·001***

FA, fatty acid; EFAD, essential fatty acid deficiency; T:T ratio, triene:tetraene (20:3*n*-9:20:4*n*-6) ratio.

*** *P* < 0·001.

† Serum fatty acid levels reported as % total fatty acids unless they are a ratio.

‡ EFAD defined as T:T ratio < 0·02.

### Correlations amongst fatty acid proportions overall and by developmental stage

**Figure 2.**
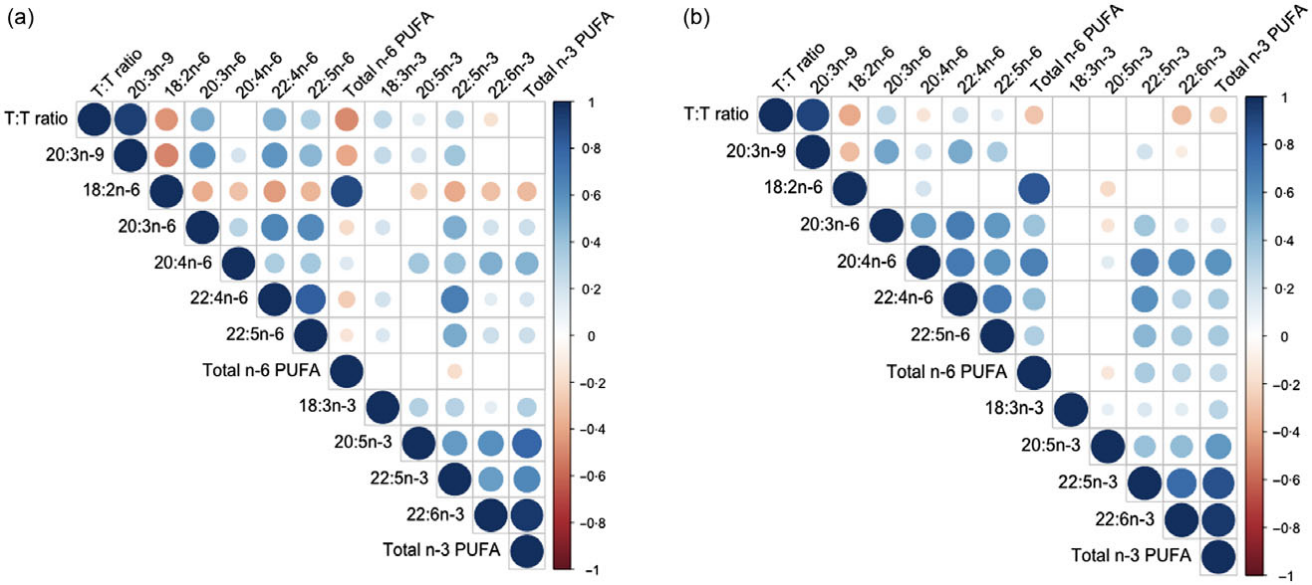
Circles represent the r correlation coefficients generated from the Pearson correlation matrix. Larger circles of deeper colours indicate a greater magnitude of association, i.e. proximity to −1 for red or 1 for blue. Only circles for coefficients with *P*-values < 0·05 are displayed. Empty boxes signify coefficients with *P*-values ≥ 0·05. T:T ratio, triene:tetraene (20:3*n*-9:20:4*n*-6) ratio; *n*-6 PUFA.

Among the whole study population, 18:2*n*-6 was negatively correlated with 20:3*n*-9 and the T:T ratio, and 20:3*n*-9 was positively correlated with other *n*-6 PUFA levels (Figure 2). Additionally, *n*-6 PUFA levels and *n*-3 PUFA levels were strongly correlated within each omega class.

In comparing children and adolescents, negative correlations between 18:2*n*-6 and *n*-6 and *n*-3 PUFA were more frequent among children than adolescents. Children also exhibited stronger positive correlations between the T:T ratio and *n*-6 and *n*-3 PUFA (Figure 2(a) *v.* (b)). Notably, shorter-chain PUFA were positively correlated with T:T ratio, and total *n*-6 PUFA were strongly negatively correlated with T:T ratio and 20:3*n*-9.

Adolescents had stronger correlations among *n*-6 PUFA levels than children, and 20:4*n*-6 was strongly positively correlated with longer chain *n*-3 PUFA and total *n*-3 PUFA (Figure 2(b) *v.* (a)). Also, 18:2*n*-6 and 20:3*n*-6 were negatively correlated with 20:5*n*-3 levels (all *P* < 0·05).

### Associations between risk factors and select PUFA levels among children

On average, 18:2*n*-6 levels were lower for children with female primary caregivers compared with those with male primary caregivers (*ß* (95 % CI): –2·63 (–4·95, –0·31) %) (Table 4). A 1-year increase in caregiver education was associated with a 0·30 % total FA increase in 18:2*n*-6 (95 % CI: (0·08, 0·51). Compared with children HUU, children PHIV had FA levels overall indicative of potential EFAD: a higher T:T ratio (0·00 (0·00, 0·00) %), 20:3*n*-9 levels (0·04 (0·01, 0·06) %), and 20:4*n*-6 levels (0·88 (0·10, 1·65) %). When looking further at IPA exposure, children PHIV with no IPA exposure drove this association (T:T ratio: 0·00 (0·00, 0·00) %; 20:3*n*-9: 0·03 (0·01, 0·05) %; 20:4*n*-6: 1·09 (0·11, 2·08) %). Children PHIV exposed to sdNVP + ZDV + 3TC had higher 22:6*n*-3 levels (0·68 (0·14, 1·22) %), but children PHIV exposed to cART had lower 18:2*n*-6 levels (–3·79 (–6·83, –0·75) %). Among children HEU, those exposed to sdNVP + ZDV + 3TC had a more favorable EFA profile with a lower T:T ratio (–0·00 (–0·00, –0·00) %) and 20:3*n*-9 levels (–0·02 (–0·04, –0·00) %).

**Table 4.** Associations between risk factors and select continuous PUFA levels among all 6–10-year-old Ugandan children

		T:T ratio		20:3*n*-9		18:2*n*-6		20:4*n*-6		18:3*n*-3		20:5*n*-3		22:6*n*-3	
Risk factor	*n*	*ß*	95 % CI	*ß*	95 % CI	*ß*	95 % CI	*ß*	95 % CI	*ß*	95 % CI	*ß*	95 % CI	*ß*	95 % CI
Child risk factor															
Age (years)^†^	243	0·00	0·00, 0·00	−0·00	−0·01, 0·01	0·21	−0·31, 0·73	0·05	−0·16, 0·26	−0·00	−0·02, 0·01	0·03	−0·01, 0·06	0·03	−0·06, 0·12
Sex^†^	243														
Male	128	Ref.	Ref.	Ref.	Ref.	Ref.	Ref.	Ref.	Ref.	Ref.	Ref.	Ref.	Ref.	Ref.	Ref.
Female	115	−0·00	−0·00, 0·00	−0·01	−0·03, 0·00	0·10	−1·42, 1·62	−0·06	−0·72, 0·60	0·03	−0·02, 0·07	0·03	−0·07, 0·12	0·11	−0·15, 0·37
Caregiver risk factor															
Caregiver sex^†^	232														
Male	19	Ref.	Ref.	Ref.	Ref.	Ref.	Ref.	Ref.	Ref.	Ref.	Ref.	Ref.	Ref.	Ref.	Ref.
Female	213	0·00	−0·00, 0·00	0·02	−0·00, 0·04	−2·63*	−4·95, –0·31	1·03	−0·13, 2·18	−0·03	−0·09, 0·03	0·05	−0·09, 0·19	0·09	−0·25, 0·43
Caregiver education (years)^†^	213	−0·00	−0·00, 0·00	−0·00	−0·00, 0·00	0·30**	0·08, 0·51	0·02	−0·07, 0·10	−0·00	−0·01, 0·00	−0·01	−0·02, 0·00	−0·03	−0·06, 0·00
HIV-related risk factors															
Perinatal HIV status^†^	243														
HUU	81	Ref.	Ref.	Ref.	Ref.	Ref.	Ref.	Ref.	Ref.	Ref.	Ref.	Ref.	Ref.	Ref.	Ref.
HEU	78	0·00	−0·00, 0·00	**−**0·00	−0·01, 0·01	0·99	−0·96, 2·94	−0·30	−1·11, 0·52	−0·02	−0·08, 0·03	−0·03	−0·13, 0·07	−0·24	−0·56, 0·09
PHIV	84	0·00**	0·00, 0·00	0·04**	0·01, 0·06	−1·69	−3·50, 0·12	0·88*	0·10, 1·65	−0·02	−0·07, 0·04	0·01	−0·11, 0·13	0·09	−0·23, 0·42
IPA exposure^‡^	241														
HUU	81	Ref.	Ref.	Ref.	Ref.	Ref.	Ref.	Ref.	Ref.	Ref.	Ref.	Ref.	Ref.	Ref.	Ref.
HEU	77														
None	37	0·00	−0·00, 0·00	−0·00	−0·02, 0·01	1·24	−1·08, 3·56	−0·93	−1·96, 0·10	−0·03	−0·09, 0·04	**−**0·09	−0·19, 0·02	−0·35	−0·77, 0·07
sdNVP 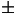 ZDV	19	0·00	−0·00, 0·00	0·00	−0·01, 0·03	1·40	−1·98, 4·78	0·31	−0·81, 1·42	−0·04	−0·15, 0·08	0·07	−0·16, 0·30	−0·36	−0·72, 0·01
sdNVP + ZDV + 3TC	8	−0·00**	−0·00, –0·00	−0·02*	−0·04, –0·00	−0·07	−2·47, 2·34	0·92	−0·39, 2·24	−0·07	−0·15, 0·00	0·10	−0·13, 0·32	0·13	−0·45, 0·72
cART	13	0·00	−0·00, 0·00	0·02	−0·02, 0·05	−0·13	−3·62, 3·37	0·42	−0·74, 1·57	0·06	−0·09, 0·20	**−**0·04	−0·16, 0·09	0·32	−0·03, 0·67
PHIV	83														
None	46	0·00**	0·00, 0·00	0·03**	0·01, 0·05	−1·08	−3·09, 0·93	1·09*	0·11, 2·08	−0·01	−0·07, 0·05	0·05	−0·12, 0·22	0·11	−0·30, 0·52
sdNVP 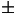 ZDV	9	0·00	−0·00, 0·02	0·08	−0·04, 0·20	−1·34	−7·08, 4·40	−0·79	−0·25, 2·01	−0·02	−0·14, 0·11	−0·10	−0·26, 0·07	−0·45	−0·93, 0·03
sdNVP + ZDV + 3TC	8	0·00	−0·00, 0·00	0·00	−0·01, 0·03	−1·92	−6·38, 2·53	0·88	−0·25, 2·01	0·05	−0·05, 0·01	0·06	−0·13, 0·24	0·68*	0·14, 1·22
cART	20	0·00	−0·00, 0·00	0·03	−0·01, 0·07	−3·79*	−6·83, –0·75	1·17	0·00, 2·33	−0·07	−0·14, 0·01	−0·08	−0·22, 0·06	0·03	−0·35, 0·40

T:T ratio, triene:tetraene (20:3*n*-9:20:4*n*-6) ratio; HUU, perinatally HIV unexposed and uninfected; HEU, perinatally HIV exposed but remained uninfected; PHIV, with perinatal HIV infection; IPA, *in utero*/peripartum antiretroviral therapy; sdNVP, single-dose nevirapine; ZDV, zidovudine; 3TC, lamivudine; cART, combination antiretroviral therapy.

*P < 0·05, **P < 0·01.

† Model adjusted for age, sex, perinatal HIV status, caregiver sex and caregiver education (thirty-nine observations were not included due to missing data for at least one covariate).

‡ Model adjusted for age, sex, IPA exposure, caregiver sex and caregiver education (thirty-nine observations were not included due to missing data for at least one covariate).

For children PHIV, those with CD4 nadir between 250–500 cells/ml had lower 20:4*n*-6 levels than those who had CD4 nadir < 250 cells/ml (*ß* (95 % CI): –1·82 (–3·51, –0·14) %) (Table 5). Regarding current ART exposure, children PHIV on DTG-based cART had higher 18:3*n*-3 levels than those cART naïve or unspecified (0·23 (0·09, 0·37) %).

**Table 5.** Associations between select continuous fatty acid levels and risk factors among 6–10-year-old Ugandan children with perinatal HIV infection (PHIV)

		T:T ratio		20:3*n*-9		18:2*n*-6		20:4*n*-6		18:3*n*-3		20:5*n*-3		22:6*n*-3	
Risk factor	*n*	*ß*	95 % CI	*ß*	95 % CI	*ß*	95 % CI	*ß*	95 % CI	*ß*	95 % CI	*ß*	95 % CI	*ß*	95 % CI
CD4 nadir^†^	74														
< 250 cells/ml	12	Ref.	Ref.	Ref.	Ref.	Ref.	Ref.	Ref.	Ref.	Ref.	Ref.	Ref.	Ref.	Ref.	Ref.
250–500 cells/ml	14	0·00	−0·00, 0·00	−0·01	−0·06, 0·04	1·71	−3·45, 6·87	−1·82*	−3·51, –0·14	0·07	−0·06, 0·20	0·03	−0·20, 0·26	0·08	−0·62, 0·77
> 500 cells/ml	48	0·00	−0·00, 0·00	−0·00	−0·04, 0·03	0·67	−3·85, 5·20	−0·61	−2·09, 0·88	0·03	−0·07, 0·13	0·18	−0·10, 0·46	0·47	−0·24, 1·17
Current ART exposure^‡^	84														
cART-naïve or unspecified	28	Ref.	Ref.	Ref.	Ref.	Ref.	Ref.	Ref.	Ref.	Ref.	Ref.	Ref.	Ref.	Ref.	Ref.
NVP	41	−0·00	−0·00, 0·00	−0·00	−0·05, 0·05	−0·07	−3·49, 3·35	0·08	−1·23, 1·39	−0·04	−0·11, 0·04	0·04	−0·16, 0·23	−0·26	−0·77, 0·25
NVP + NRTI	11	0·00	−0·00, 0·00	−0·00	−0·07, 0·06	2·91	−2·21, 8·03	−1·38	−3·82, 1·06	−0·05	−0·15, 0·05	−0·03	−0·21, 0·15	−0·24	−0·80, 0·32
DTG	4	0·00	−0·00, 0·01	0·04	−0·02, 0·10	−2·41	−6·89, 2·06	−1·35	−3·81, 1·11	0·23**	0·09, 0·37	0·22	−0·80, 0·53	−0·11	−0·77, 0·55

T:T ratio, triene:tetraene (20:3*n*-9:20:4*n*-6) ratio; ART, antiretroviral therapy; NVP, nevirapine; NRTI, nucleoside RT inhibitor; DTG, dolutegravir.

*P < 0·05, **P < 0·01.

† Model 1 adjusted for age, sex, CD4 nadir, caregiver sex, and caregiver education (eighteen observations were not included due to missing data for at least one covariate).

‡ Model 2 adjusted for age, sex, current ART exposure, caregiver sex and caregiver education (thirteen observations were not included due to missing data for at least one covariate).

### Associations between risk factors and select PUFA levels among adolescents

Among adolescents, a 1-year increase in age was associated with greater EFA sufficiency, as indicated by a lower T:T ratio (*ß* (95 % CI): –0·00 (–0·00, –0·00) %) and 20:3*n*-9 levels (–0·01 (–0·01, –0·00) %) and higher 18:2*n*-6 (0·13 (0·02, 0·24) %), 20:4*n*-6 (0·05 (0·00, 0·10) %), and 22:6*n*-3 levels (0·03 (0·00, 0·06) %) (Table 6). A 1-year increase in caregiver education, however, was associated with decreased 22:6*n*-3 levels (–0·02 (–0·04, –0·01) %). Regarding perinatal HIV status, adolescents PHIV had lower 20:5*n*-3 levels (–0·05 (–0·09, –0·02) %) than adolescents HUU. In terms of IPA exposure, adolescents PHIV with no exposure had lower EFA and metabolite levels (18:2*n*-6: –1·00 (–1·81, –0·20) %; 20:5*n*-3: –0·06 (–0·10, –0·02) %). Adolescents PHIV exposed to sdNVP 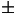 ZDV had a lower T:T ratio (–0·02 (–0·03, –0·01) %), 20:3*n*-9 levels (–0·07 (–0·10, –0·03) %) and 20:5*n*-3 levels (–0·07 (–0·12, –0·02) %), which overall indicates higher EFA sufficiency. However, adolescents PHIV with cART exposure had lower 18:3*n*-3 (–0·05 (–0·08, –0·01) %) and 20:5*n*-3 (–0·06 (–0·11, –0·02) %) levels. Adolescents HEU exposed to sdNVP + ZDV + 3TC had a lower risk of EFAD compared with adolescents HUU as indicated by higher 20:4*n*-6 levels (0·59 (0·17, 1·00) %). In contrast, adolescents HEU exposed to cART had lower 22:6*n*-3 levels (–0·27 (–0·52, –0·02) %), which may indicate potential EFAD.

**Table 6.** Associations between select continuous fatty acid levels and risk factors among all 11–18-year-old Ugandan adolescents

		T:T ratio		20:3*n*-9		18:2*n*-6		20:4*n*-6		18:3*n*-3		20:5*n*-3		22:6*n*-3	
Risk factor	*n*	*ß*	95 % CI	*ß*	95 % CI	*ß*	95 % CI	*ß*	95 % CI	*ß*	95 % CI	*ß*	95 % CI	*ß*	95 % CI
Adolescent risk factor															
Age (years)^†^	382	−0·00**	−0·00, –0·00	−0·01**	−0·01, –0·00	0·13*	0·02, 0·24	0·05*	0·00, 0·10	0·00	−0·00, 0·01	0·00	−0·00, 0·01	0·03*	0·00, 0·06
Sex^†^	383														
Male	171	Ref.	Ref.	Ref.	Ref.	Ref.	Ref.	Ref.	Ref.	Ref.	Ref.	Ref.	Ref.	Ref.	Ref.
Female	212	−0·00	−0·01, 0·00	−0·02	−0·05, 0·01	−0·64*	−1·19, –0·08	−0·03	−0·26, 0·21	0·01	−0·02, 0·04	0·01	−0·01, 0·04	0·17**	0·05, 0·30
Caregiver risk factor															
Caregiver sex^†^	364														
Male	49	Ref.	Ref.	Ref.	Ref.	Ref.	Ref.	Ref.	Ref.	Ref.	Ref.	Ref.	Ref.	Ref.	Ref.
Female	315	−0·01	−0·02, 0·00	−0·04	−0·09, 0·00	−0·02	−0·99, 0·95	−0·05	−0·36, 0·25	−0·01	−0·06, 0·03	−0·01	−0·05, 0·03	−0·12	−0·30, 0·06
Caregiver education (years)^†^	358	0·00	−0·00, 0·00	0·00	−0·00, 0·00	−0·04	−0·12, 0·03	−0·03	−0·06, 0·00	−0·00	−0·01, 0·00	−0·00	−0·01, 0·00	−0·02*	−0·04, –0·01
HIV-related risk factors															
Perinatal HIV status^†^	383														
HUU	124	Ref.	Ref.	Ref.	Ref.	Ref.	Ref.	Ref.	Ref.	Ref.	Ref.	Ref.	Ref.	Ref.	Ref.
HEU	133	0·00	−0·00, 0·01	0·02	−0·01, 0·06	−0·14	−0·85, 0·58	0·11	−0·20, 0·42	−0·01	−0·04, 0·03	−0·02	−0·05, 0·02	−0·24	−0·56, 0·09
PHIV	126	0·00	−0·00, 0·01	0·02	−0·01, 0·05	−0·60	−1·27, 0·08	0·10	−0·17, 0·37	0·01	−0·03, 0·05	−0·05**	−0·09, –0·02	0·09	−0·23, 0·42
IPA exposure^‡^	383														
HUU	124	Ref.	Ref.	Ref.	Ref.	Ref.	Ref.	Ref.	Ref.	Ref.	Ref.	Ref.	Ref.	Ref.	Ref.
HEU															
None	57	0·01	−0·00, 0·02	0·04	−0·01, 0·09	−0·57	−1·36, 0·22	−0·03	−0·43, 0·36	0·02	−0·03, 0·07	**−**0·03	−0·06, 0·01	−0·13	−0·33, 0·07
sdNVP 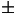 ZDV	26	0·00	−0·01, 0·02	0·01	−0·05, 0·07	0·12	−1·00, 1·25	0·02	−0·46, 0·50	−0·01	−0·05, 0·03	−0·00	−0·08, 0·07	−0·21	−0·47, 0·05
sdNVP + ZDV + 3TC	26	−0·00	−0·01, 0·00	0·00	−0·04, 0·04	0·43	−0·65, 1·51	0·59**	0·17, 1·00	−0·04	−0·09, 0·01	0·01	−0·08, 0·09	0·10	−0·17, 0·36
cART	24	0·00	−0·01, 0·02	0·02	−0·04, 0·09	0·07	−1·13, 1·28	0·07	−0·54, 0·67	−0·01	−0·05, 0·03	**−**0·03	−0·06, 0·01	−0·27*	−0·52, –0·02
PHIV															
None	64	0·01	−0·00, 0·02	0·03	−0·00, 0·07	−1·00*	−1·81, –0·20	−0·08	−0·42, 0·27	0·04	−0·02, 0·10	−0·06**	−0·10, –0·02	−0·18	−0·38, 0·02
sdNVP 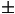 ZDV	12	−0·02**	−0·03, –0·01	−0·07***	−0·10, –0·03	0·54	−0·53, 1·61	−0·00	−0·44, 0·44	−0·03	−0·09, 0·02	−0·07**	−0·12, –0·02	−0·01	−0·28, 0·26
sdNVP + ZDV + 3TC	13	0·01	−0·01, 0·02	0·05	−0·01, 0·11	−1·15	−2·65, 0·35	0·53	−0·10, 1·16	0·03	−0·12, 0·18	0·06	−0·18, 0·29	0·25	−0·15, 0·64
cART	37	−0·00	−0·01, 0·01	0·01	−0·04, 0·07	−0·06	−1·05, 0·93	0·33	−0·65, 0·72	−0·05**	−0·08, –0·01	−0·06**	−0·11, –0·02	0·11	−0·17, 0·38

T:T ratio, triene:tetraene (20:3*n*-9:20:4*n*-6) ratio; HUU, perinatally HIV unexposed and uninfected; HEU, perinatally HIV exposed but remained uninfected; PHIV, with perinatal HIV infection; IPA, *in-utero*/peripartum antiretroviral therapy; sdNVP, single-dose nevirapine; ZDV, zidovudine; 3TC, lamivudine; cART, combination antiretroviral therapy.

*P < 0·05, **P < 0·01, ***P < 0·001.

† Model adjusted for age, sex, perinatal HIV status, caregiver sex and caregiver education (forty-three observations were not included due to missing data for at least one covariate).

‡ Model adjusted for age, sex, IPA exposure, caregiver sex and caregiver education (forty-three observations were not included due to missing data for at least one covariate).

Among adolescents PHIV, lower CD4 nadir was associated with higher potential EFAD. Specifically, T:T ratio was lower for adolescents PHIV CD4 nadir > 500 cells/ml (*ß* (95 % CI): –0·02 (–0·03, –0·00) %) than those with CD4 nadir < 250 cells/ml (Table 7). Additionally, 22:6*n*-3 levels were higher for adolescents with CD4 nadir between 250 and 500 cells/ml (0·33 (0·04, 0·63) %) and CD4 nadir > 500 cells/ml (0·39 (0·14, 0·64) %), and 20:4*n*-6 levels were higher for adolescent PHIV whose CD4 nadir was > 500 cells/ul (0·71 (0·20, 1·22) %). Adolescents PHIV currently on cART showed greater EFA sufficiency than adolescents PHIV cART naïve or unspecified; those on NVP + NRTI-based cART had higher 18:2*n*-6 levels (2·33 (0·65, 4·01) %), and those on DTG-based cART had a lower T:T ratio (–0·01 (–0·03, –0·00) %) and higher 22:6*n*-3 levels (0·57 (0·30, 0·83) %).

**Table 7.** Associations between select fatty acid quartiles and risk factors among 11–18-year-old Ugandan adolescents with perinatal HIV infection (PHIV)

		T:T ratio		20:3*n*-9		18:2*n*-6		20:4*n*-6		18:3*n*-3		20:5*n*-3		22:6*n*-3	
Risk factor	*n*	*ß*	95 % CI	*ß*	95 % CI	*ß*	95 % CI	*ß*	95 % CI	*ß*	95 % CI	*ß*	95 % CI	*ß*	95 % CI
CD4 nadir^†^	108														
< 250 cells/ml	17	Ref.	Ref.	Ref.	Ref.	Ref.	Ref.	Ref.	Ref.	Ref.	Ref.	Ref.	Ref.	Ref.	Ref.
250–500 cells/ml	41	−0·01	−0·02, 0·01	−0·02	−0·08, 0·05	0·14	−1·38, 1·66	0·42	−0·13, 0·97	0·05	−0·05, 0·15	0·04	−0·04, 0·12	0·33*	0·04, 0·63
> 500 cells/ml	50	−0·02*	−0·03, –0·00	−0·05	−0·11, 0·02	0·59	−0·92, 2·10	0·71**	0·20, 1·22	0·03	−0·09, 0·14	0·02	−0·03, 0·06	0·39**	0·14, 0·64
Current ART exposure^‡^	122														
cART-naïve or unspecified	32	Ref.	Ref.	Ref.	Ref.	Ref.	Ref.	Ref.	Ref.	Ref.	Ref.	Ref.	Ref.	Ref.	Ref.
NVP	11	−0·00	−0·00, 0·00	0·01	−0·07, 0·07	−0·31	−2·01, 1·38	0·74	−0·26, 1·75	−0·05	−0·14, 0·05	0·14	−0·10, 0·39	0·26	−0·31, 0·82
NVP + NRTI	6	0·00	−0·00, 0·00	−0·03	−0·08, 0·03	2·33**	0·65, 4·01	0·08	−0·67, 0·08	0·03	−0·22, 0·27	−0·07	−0·17, 0·04	−0·26	−0·68, 0·15
Kaletra	70	−0·01	−0·02, 0·01	−0·01	−0·06, 0·04	0·61	−0·46, 1·67	0·04	−0·40, 0·46	0·01	−0·09, 0·11	0·00	−0·03, 0·04	0·08	−0·22, 0·38
DTG	3	−0·01*	−0·03, –0·00	−0·04	−0·10, 0·03	1·77	−0·43, 3·98	0·23	−1·43, 1·90	−0·07	−0·17, 0·03	0·32	−0·13, 0·78	0·57***	0·30, 0·83

T:T ratio, triene:tetraene (20:3*n*-9:20:4*n*-6) ratio; ART, antiretroviral therapy; NVP, nevirapine; NRTI, nucleoside RT inhibitor; DTG, dolutegravir.

*P < 0·05, **P < 0·01, ***P < 0·001.

† Model 1 adjusted for age, sex, CD4 nadir, caregiver sex and caregiver education (thirty-two observations were not included due to missing data for at least one covariate).

‡ Model 2 adjusted for age, sex, current ART exposure, caregiver sex and caregiver education (twenty-one observations were not included due to missing data for at least one covariate).

## Discussion

In this sample of children and adolescents from Uganda, the FA profile was more indicative of EFAD in adolescents relative to children. Additionally, adolescents were more likely to be stunted or at risk of stunting. From a dietary perspective, these findings may reflect a higher intake of processed, fatty foods and a decreased intake of lean animal products, legumes, seed oils and fish. Research from boarding schools in Kampala suggests that adolescents are more likely to eat fast food and wheat-based snacks than legumes and fish even when these PUFA-rich foods are available^(27)^. Teachers in these schools have observed these trends and note that adolescents in boarding school generally make poorer dietary choices, as their food decisions are largely autonomous^(27)^. Studies from the USA and UK show that adolescent food choices are more highly driven by social experience, convenience and autonomy than by food itself^(40,41)^, which further supports the westernisation of the Ugandan diet and highlights Ugandan adolescents as particularly vulnerable to low PUFA intake. Of note, child chronologic age was associated with increased *n*-6 and *n*-3 PUFA levels among adolescents but not children. This may reflect adolescents’ evolving nutritional habits as they receive more nutrition education in boarding school and become more health conscious^(27)^, but this disagrees with studies which show that dietary behaviours get worse as adolescents transition into young adulthood^(42)^. Overall, this observation suggests that although FA profile is worse among Ugandan adolescents, FA profile may improve with increasing age.

Moreover, Ugandan adolescents may be more prone to low PUFA levels than children due to FA metabolism alterations that occur during adolescence. Specifically, the hormone changes associated with adolescence, i.e. increased insulin resistance and increased estrogen/testosterone release, may impact the activity of Δ-6 and Δ-5 desaturases, which are necessary for EFA metabolism^(43)^. Translational studies demonstrated increased Δ-6 activity and decreased Δ-5 activity with increased insulin resistance^(44)^. Additionally, estrogen increased and testosterone decreased Δ-5 and Δ-6 activity in rats^(45,46)^. These metabolic changes may in turn decrease the levels of EFA and their longest-chain metabolites and increase their intermediate metabolites among adolescents, with further modification according to biological sex, in adolescents *v.* children. These changes are partially supported by the findings in this study, as adolescents exhibited lower 18:2*n*-6, higher intermediate *n*-6 PUFA, lower 20:4*n*-6, lower 20:5*n*-3 and lower 22:6*n*-3 than children. Thus, dietary and hormonal factors may drive the differences in FA levels by developmental stage.

Consistent with the study hypothesis, perinatal HIV infection status was associated with a PUFA profile indicative of EFAD. These results support previous studies among this 6–10-year-old study population, which demonstrated higher *n*-6 PUFA levels (besides 18:2*n*-6) and 20:3*n*-9 and T:T ratio levels among youth PHIV relative to youth HUU^(7)^. The implications of potential higher EFAD prevalence for the overall health and well-being of youth PHIV are many. EFAD may contribute to and/or exacerbate immune dysregulation^(4,8)^, stunting^(6,7)^, and adverse cognitive development^(5,8)^ among youth PHIV.

Among youth affected by perinatal HIV, observed PUFA levels further varied according to IPA regimen. Specifically, in both the children and adolescents PHIV and HEU, lack of IPA exposure and cART IPA exposure was associated with a FA profile suggestive of EFAD. On the other hand, youth PHIV and HEU exposed to sdNVP ± ZDV and sdNVP + ZDV + 3TC exhibited higher EFA sufficiency, which partially supports previous findings^(24,25)^. These complex associations among youth PHIV and HEU must be interpreted in the context of the prevailing standard of care for IPA access during their era of birth. Specifically, youth whose mothers received cART during pregnancy were more likely to have more severe HIV disease and worse overall health^(34,35)^. While HIV disease severity complicates the interpretation of these associations, additional research is needed to determine whether IPA exposure among youth PHIV and HEU is associated with long-lasting alterations in the absorption and metabolic processing of EFA. In sum, it is important for future studies to replicate and clarify observations reported herein and inform knowledge gaps with respect to the long-term relevance of IPA exposure for FA profile and health outcomes among youth HEU and PHIV.

Beyond IPA exposure, current HIV treatment parameters, such as CD4 nadir and current ART regimen, demonstrated significant correlations with FA profile among youth PHIV. Adolescents PHIV displayed positive associations between EFA sufficiency and CD4 nadir, while children PHIV displayed negative associations. These results partially support findings from a study of 10–18-year-old Ugandan youth that demonstrated a negative association between PUFA and activated CD4 + T cells^(47)^, as well as findings from a study among Ugandan adults PHIV that showed a positive association between *n*-6 PUFA and CD4 + T cell counts^(48)^. Further research among younger children is needed to examine the variation by developmental stage. Moreover, the findings among youth PHIV using DTG-based cART suggest this regimen is correlated with higher EFA sufficiency. These results support the overall consensus that DTG is a preferred ART drug due to its effectiveness in decreasing virology and adverse events^(49)^, notably among paediatric patients^(50)^. Ultimately, higher CD4 nadir and NVP + NRTI- and DTG-based ART regimens were associated with increased beneficial PUFA levels among adolescents, but these relationships need to be further studied. Collectively, these findings highlight the continued importance of optimal HIV disease management to prevent EFAD in youth affected by HIV and may suggest that HIV-specific pathology is an alternative explanation for IPA-related variations in FA levels observed.

There were several strengths of this study. First, it has novel objectives, as risk factors related to FA levels among youth populations affected by HIV are understudied. Another strength is the robust nature of the statistical analysis that included control for a range of confounding variables and clustering within households. Additionally, all HIV-related risk factor information was obtained objectively through medical records, ensuring that there was little misclassification based on perinatal HIV status and IPA exposure in this study of children and adolescents. IPA exposure was also stratified by perinatal HIV status and used HUU as the reference group, which led to the informative investigation of the differential effects of IPA by perinatal HIV status. Finally, the sample size was relatively large, providing sufficient statistical power.

Several limitations must also be considered in the interpretation of our findings. Dietary intake information was not obtained from the study population, so FA levels could not be examined in relation to dietary intake. Moreover, GC/MS analysis of the child and adolescent serum samples were done separately, which may result in variation of the results. However, study personnel ran quality check samples of known concentration during both batch runs to ensure proper quantification. The study design was cross-sectional, and the observational nature of the study implies that residual confounding cannot be excluded as an alternative explanation for the findings described. Regarding IPA exposure, study personnel could not discern differences in the timing of ART initiation, which introduces the immortal time bias. Also, some of the IPA exposure categories were relatively small, so there was limited power to detect IPA exposure associations with FA levels. Finally, these results lack generalisability, as they can only be applied to a specific study population (6–18-year-old Ugandan youth living in Kampala with varying levels of perinatal HIV status).

In conclusion, FA levels significantly differed by developmental stage, with adolescents having lower proportions of *n*-6 and *n*-3 PUFA. Overall, youth PHIV had PUFA profiles more indicative of EFAD than youth HUU, but these associations ultimately differed by IPA exposure type and developmental stage. EFA sufficiency among youth HEU was also correlated with IPA exposure and varied by developmental stage. Altogether, these results suggest the importance of studying the long-term health impacts of factors related to perinatal HIV status, notably IPA exposure, in children and adolescents affected by, not just infected with, HIV. Future research should focus on elucidating the contributory role of IPA exposure type to FA level differences and identifying the implications of FA level differences based on perinatal HIV-related risk factors on growth and cognitive development outcomes in youth HEU and PHIV.
